# Anthocyanins and Their Variation in Red Wines I. Monomeric Anthocyanins and Their Color Expression

**DOI:** 10.3390/molecules17021571

**Published:** 2012-02-07

**Authors:** Fei He, Na-Na Liang, Lin Mu, Qiu-Hong Pan, Jun Wang, Malcolm J. Reeves, Chang-Qing Duan

**Affiliations:** 1 Center for Viticulture and Enology, College of Food Science & Nutritional Engineering, China Agricultural University, Beijing, 100083, China; 2 Faculty of Applied Science, Business and Computing, Eastern Institute of Technology, Napier 4142, New Zealand

**Keywords:** monomeric anthocyanin, red wine, self-association, copigmentation, degradation, enology

## Abstract

Originating in the grapes, monomeric anthocyanins in young red wines contribute the majority of color and the supposed beneficial health effects related to their consumption, and as such they are recognized as one of the most important groups of phenolic metabolites in red wines. In recent years, our increasing knowledge of the chemical complexity of the monomeric anthocyanins, their stability, together with the phenomena such as self-association and copigmentation that can stabilize and enhance their color has helped to explain their color representation in red wine making and aging. A series of new enological practices were developed to improve the anthocyanin extraction, as well as their color expression and maintenance. This paper summarizes the most recent advances in the studies of the monomeric anthocyanins in red wines, emphasizing their origin, occurrence, color enhancing effects, their degradation and the effect of various enological practices on them.

## 1. Introduction

Anthocyanins, also known as anthocyans, are water soluble flavonoid pigments that, depending on pH, and in some cases complexing agents, can contribute diverse colors such as red, purple and blue [1,2]. They are widely spread throughout the plant kingdom, and they can occur in almost all tissues of higher plants, including roots, stems, leaves, flowers, and fruits [3]. Consequently they are considered to be a group of the major natural pigments in the plant-derived food, including red wines [1,4].

Color is one of the most important attributes of red wines, and the principal sources of red color in wines come from the anthocyanins or their further derivatives that are extracted or formed during the vinification process [1,5]. The typical concentrations of free anthocyanins in full-bodied young red wines are around 500 mg/L, but can in some cases be higher than 2,000 mg/L [6,7,8,9]. Normally, anthocyanins are mainly located in the grape skins, with a few exceptions, in the so-called “teinturier” grapes, which have anthocyanins in both of the skin and the pulp [10,11]. During fermentation and, especially in the first one or two years of maturation, the monomeric anthocyanins in wines undergo a wide variety of reactions and associations and various anthocyanin-derived new pigments are formed, which are extremely crucial for the color stability [4,12,13]. Consequently, although the concentration of monomeric anthocyanins in red wines declines constantly, red wines can still maintain an essentially red color. The reactions and associations involve complex mechanisms, including relatively short-term ones, such as self-association and copigmentation, and the relatively long-term ones, such as the formation of polymeric anthocyanins with flavan-3-ols and proanthocyanidins, as well as the formation of new pigments, such as pyranoanthocyanins and their further polymerized products [4,12,13].

Unlike other flavonoid compounds in red wines, anthocyanins do not intrinsically contribute astringency or bitterness to the mouth feel. [14,15]. Though anthocyanins are odorless and nearly flavorless, they can interact with some aroma substances and influence wine flavor [16]. Furthermore, numerous studies have revealed the potential pharmacological properties of anthocyanins and their derived compounds in red wines on human health [17]. Such benefits mainly include free radical scavenging and antioxidant activity, protective effects against UV irradiation and on cardiovascular health, anticancer and antimutagenic activity [18,19,20,21,22,23,24,25,26]. However, these beneficial health effects of anthocyanins are still a controversial issue. Until now, the majority of the related researches were only carried out *in vitro* and their conclusions are not robust enough. In fact, nowadays one of the principal challenges is the need for better-designed clinical studies to improve the current knowledge and elucidate their real effects on human health [26]. Nevertheless, the potential use of anthocyanins as natural colorants to substitute for synthetic dyes in the food industry may also have many potential benefits [27]. As a result, a lot of attention has been paid to the extraction of anthocyanins from grape skins and pomace produced during winemaking [28,29,30,31].

With the help of modern chromatography techniques [such as high-performance or high-pressure liquid chromatography (HPLC) and high-speed countercurrent chromatography (HSCCC)] and electrophoresis [such as capillary zone electrophoresis (CZE)] methods, all of the simple anthocyanins, and their derivatives in red wines can be separated quickly and efficiently [32,33,34,35,36]. In addition, by using modern qualitative technologies such as mass spectrometry (MS) and nuclear magnetic resonance (NMR), most of these pigments can be identified correctly and effectively [37,38]. With the help of such techniques, the monomeric anthocyanin profiles of young red wines from various grape varieties, the structures of different forms of monomeric anthocyanins in the equilibria of red wines, the effect of self-association and copigmentation interaction have been verified [12,39]. In recent years, near-infra-red (NIR) and mid-infra-red (MIR) spectrometry were applied to the analysis of the content of monomeric anthocyanins in grape berries or wines without the destructions of the samples, which can really facilitate the determination of adulterations and the prediction of their color evolution during red wine making [40,41,42].

The aim of this paper is to summarize both of the basic knowledge and the newest achievements in the field of monomeric anthocyanins in red wines. We also hope this paper will be a valuable reference resource, which can provide beneficial inspiration for the future research in this exciting and rapidly expanding field.

## 2. Free Anthocyanins in Wines

### 2.1. Free Anthocyanins

In young red wines, free anthocyanins are the principal source of red color, though monomeric anthocyanins are not particularly stable. As red grapes are the exclusive source of these monomeric anthocyanins, their composition determines the composition of the anthocyanin profile of the corresponding red wines automatically and significantly [7,9,43,44,45,46,47]. These monomeric or free anthocyanins are gradually incorporated into their derived pigments, including copigments and polymeric pigments involving other phenolics during wine aging, contributing to a progressive shift of the red-purple color of young red wines towards the more red-orange color of aged red wines [12].

**Figure 1:**
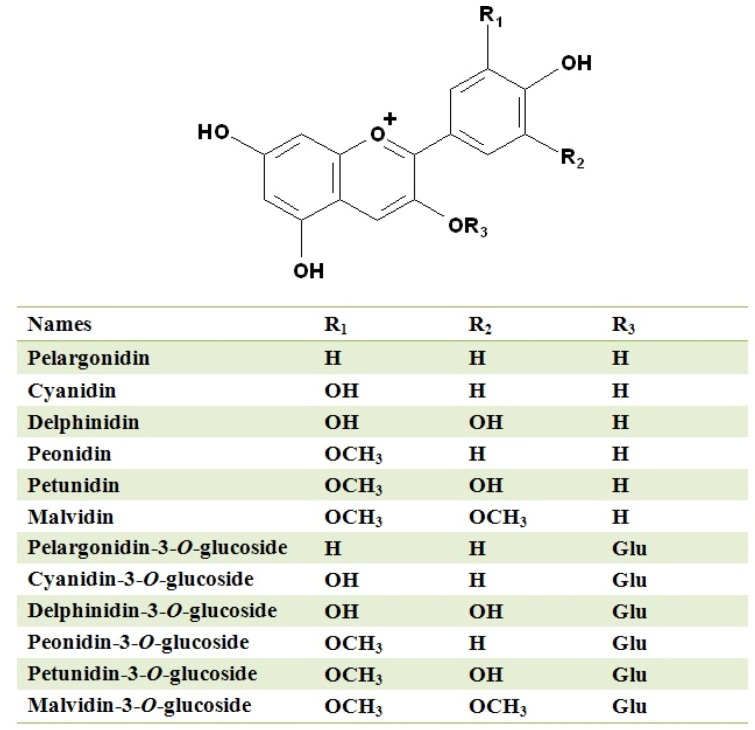
Structures of the monomeric anthocyanins naturally occurring in *Vitis vinifera* wines and their corresponding anthocyanidins [12].

Normally, in the red wines which are made from *V. vinifera* grapes, the main monomeric anthocyanins are the 3-*O*-monoglucosides of the six free anthocyanidins, including pelargonidin-3-*O*-glucoside (callistephin), cyanidin-3-*O*-glucoside (kuromanin), delphinidin-3-*O*-glucoside (myrtillin), peonidin-3-*O*-glucoside (peonin), petunidin-3-*O*-glucoside (petunin) and malvidin-3-*O*-glucoside (oenin) [1,12,48]. Their structures are illustrated in Figure 1. Such anthocyanidins differ from each other by the number and position of the hydroxyl and methoxyl substituent groups in the B ring of the molecule. The hydroxylation pattern of the anthocyanins in the B ring can directly affect the hue and color stability due to the effect on the delocalized electrons path length in the molecule. For example, the anthocyanins with more hydroxyl groups in the B rings can contribute more blueness, whereas the degree of methylation of the B rings can increase the redness. Thus, the malvidin-3-*O*-glucoside and its derivates are the reddest anthocyanins [12,49]. Among these monomeric anthocyanins, malvidin-3-*O*-glucoside and its derivatives are usually the most abundant and are the source of most of the red color in very young red wines, varying from more than 90% in Grenache to just less than 50% in Sangiovese, whereas pelargonidin-3-*O*-glucoside is difficult to detect because of its low levels [12,48,50,51]. In one more recent study, malvidin-3-*O*-glucoside only accounted for no more than 42% of the total anthocyanins in a Merlot wine in the end of alcoholic fermentation [52]. Furthermore, by using more sensitive analytical techniques, some anthocyanidin-3,5-*O*-diglucosides have also been found in trace amounts in some red grapes or wines from certain *V. vinifera* varieties [53,54,55]. Recently it has been suggested that even 3,7-*O*-diglucoside anthocyanins are present [56].

**Figure 2:**
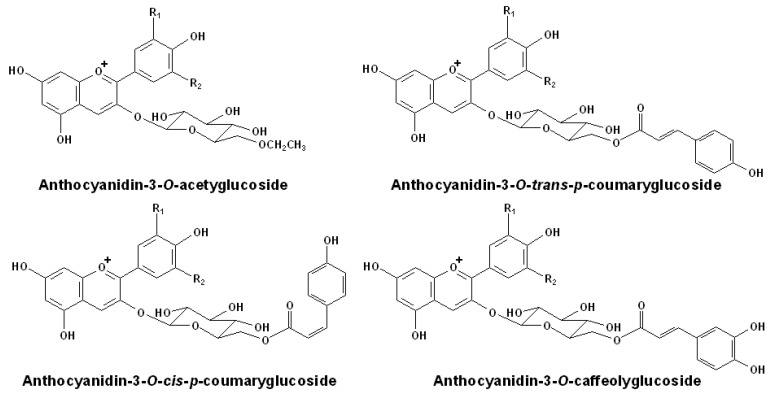
Structures of normal acetylated anthocyanins in red wines [57,58,59,60].

Besides, there is also a series of acylated anthocyanins in red grapes, including the aliphatic acetyl and the aromatic *p*-coumaroyl, caffeoyl that occur at the C6″ position of the glucose moiety [57,58,59]. Interestingly, even the 3-*O*-(6-*p*-coumaroyl)-glucosides of the same anthocyanidin can have two different stereoisomerism structures, the *cis-* and *trans-* isomers [60]. The structures of normal acetylated anthocyanins found in red wines are illustrated in Figure 2.

Recently, some new anthocyanins acetylated with unusual organic acids, such as lactic acid and ferulaic acid, were also identified in trace amounts in some red wines [56]. The acylation of the above mentioned anthocyanins can increase both their stability and solubility. However, not all the *V. vinifera* red varieties, such as ‘Pinot noir’ and red-colored mutants of the white grape varieties, contain acylated anthocyanins [61,62]. Moreover, the amount of acylated anthocyanins is highly variable according to the grape variety [57,58,59,60,61,62]. Some other grape species, such as muscadine grapes (*V. rotunidfolia*) also do not accumulate acylated anthocyanins [63].

**Table 1:** The mass spectral and UV-vis data of the major free anthocyanins in various red wines that made from *V. vinifera* grapes [76,77,78,79,80,81].

Compounds	Molecular ion M^+^ (*m/z*)	Fragment ion M^+^ (*m/z*)	λ_max_ (nm)
Delphinidin-3-*O*-monoglucoside	465	303	523
Cyanidin-3-*O*-monoglucoside	449	287	515
Petunidin-3-*O*-monoglucoside	479	317	526
Peonidin-3-*O*-monoglucoside	463	301	515
Malvidin-3-*O*-monoglucoside	493	331	530
Pelargonidin-3-*O*-monoglucoside	433	271	505
Delphinidin-3-*O*-acetylglucoside	507	303,465	521
Cyanidin-3-*O*-acetylglucoside	491	287,449	514
Petunidin-3-*O*-acetylglucoside	521	317,479	530
Peonidin-3-*O*-acetylglucoside	505	301,463	518
Malvidin-3-*O*-acetylglucoside	535	331,493	521
Pelargonidin-3-*O*-acetylglucoside	475	271,433	Unknown
Delphinidin-3-*O*-coumaroylglucoside	611	303,465	530
Cyanidin-3-*O*-coumaroylglucoside	595	287,449	522
Petunidin-3-*O*-coumaroylglucoside	625	317,479	531
Peonidin-3-*O*-coumaroylglucoside	609	301,463	523
Malvidin-3-*O*-coumaroylglucoside	639	331,493	521
Pelargonidin-3-*O*-coumaroylglucoside	579	271,433	Unknown
Delphinidin-3-*O*-caffeoylglucoside	627	303,465	Unknown
Cyanidin-3-*O*-caffeoylglucoside	611	287,449	Unknown
Petunidin-3-*O*-caffeoylglucoside	641	317,479	Unknown
Peonidin-3-*O*-caffeoylglucoside	625	301,463	525
Malvidin-3-*O*-caffeoylglucoside	655	331,493	538
Pelargonidin-3-*O*-caffeoylglucoside	595	271,433	Unknown
Malvidin-3-*O*-feurlylglucoside	669	331,493	532

Therefore, the category, the proportion and amount of anthocyanins in red grapes largely depends on the grape varieties and the growing conditions, such as viticulture practices and the weather regional characteristics [43,47,64,65]. The anthocyanin profiles of grape skins can be used as chemotaxonomy criteria to distinguish grape varieties or even the colons, since it is proposed that the relationship between the individual or total concentration of different anthocyanins can represent varietal characterization [47,66,67,68]. However, the anthocyanin composition in red wines depends not only on the original anthocyanin profile in grape berries, but also on the winemaking techniques employed [69]. Furthermore, the monomeric anthocyanins in red wines are not particularly stable and are easily oxidized [70]. Such anthocyanins decrease significantly with aging, with a concomitant increase in condensed products [1,4,5,12,13,50,71]. For example, the acylated anthocyanins disappear rapidly within a few months after fermentation. The concentration of these monomeric anthocyanins vary a lot during barrel and bottle aging, and they only contribute to the red color in relatively young red wines [4,71]. In fact, most of these free anthocyanins will combine or condense with other phenolic compounds in red wines to form more complex and stable pigments, while a relatively small fraction disappears by degradation, oxidation, precipitation, or formation of other colorless compounds, such as castavinols which can act as a reserve of anthocyanins [12,50,72]. Thus, it is not appropriate to use the profiles of monomeric anthocyanins in red wines to identify the grape varieties that were used in the corresponding winemaking, especially in aged wines, but previous reports usually gave the positive answers [47,69,73,74,75]. All the detailed information of the normal monomeric anthocyanins that can be detected in young red wines that made from *V. vinifera* grapes is summarized in Table 1, and these include MS and UV-vis absorption data.

On the other hand, in red wines which are made from non-*V. vinifera* grapes, both 3-*O*-monoglucoside and 3,5-*O*-diglucoside of anthocyanins can be present, including pelargonidin-3,5-*O*-diglucoside (pelargonin), cyanidin-3,5-*O-*diglucoside (cyanin), delphinidin-3,5-*O*-diglucoside, peonidin-3,5-*O*-diglucoside, petunidin-3,5-*O*-diglucoside and malvidin-3,5-*O*-diglucoside (malvin) and their corresponding acylated anthocyanins [82,83,84,85,86,87]. Normally, anthocyanin diglucosides are more stable than their monoglucoside counterparts, but are more susceptible to browning and are less colored [12,88,89,90,91]. Table 2 presents detailed information on the monomeric diglucosidic anthocyanins in young red wines made from non-*V. vinifera* and hybrid grapes, as shown below.

**Table 2:** The mass spectral and UV-vis data of the major diglucosidic free anthocyanins in various red wines that made from non-*V. vinifera* grapes [79,85,86,87].

Compounds	Molecular ion M^+^(*m/z*)	Fragment ion M^+^ (*m/z*)	λ_max_ (nm)
Delphinidin-3,5-*O*-diglucoside	627	303,465	520
Cyanidin-3,5-*O*-diglucoside	611	287,449	516
Petunidin-3,5-*O*-diglucoside	641	317,479	523
Peonidin-3,5-*O*-diglucoside	625	301,463	513
Malvidin-3,5-*O*-diglucoside	655	331,493	524
Pelargonidin-3,5-*O*-diglucoside	595	271,433	Unknown
Delphinidin-3-*O*-acetylglucoside-5-*O*-glucoside	669	303,465,507	Unknown
Cyanidin-3-*O*-acetylglucoside-5-*O*-glucoside	653	287,449,611	516
Petunidin-3-*O*-acetylglucoside-5-*O*-glucoside	683	317,479,641	530
Peonidin-3-*O*-acetylglucoside-5-*O*-glucoside	667	301,463,625	Unknown
Malvidin-3-*O*-acetylglucoside-5-*O*-glucoside	697	331,493,655	530
Pelargonidin-3-*O*-acetylglucoside-5-*O*-glucoside	637	271,433,595	Unknown
Delphinidin-3-*O*-coumaroylglucoside-5-*O*-glucoside	773	303,465,627	530
Cyanidin-3-*O*-coumaroylglucoside-5-*O*-glucoside	757	287,449,611	524
Petunidin-3-*O*-coumaroylglucoside-5-*O*-glucoside	787	317,479,641	530
Peonidin-3-*O*-coumaroylglucoside-5-*O*-glucoside	771	301,463,625	520
Malvidin-3-*O*-coumaroylglucoside-5-*O*-glucoside	801	331,493,655	530
Pelargonidin-3-*O*-coumaroylglucoside-5-*O*-glucoside	741	271,433,595	Unknown
Delphinidin-3-*O*-caffeoylglucoside-5-*O*-glucoside	789	303,465,507	Unknown
Cyanidin-3-*O*-caffeoylglucoside-5-*O*-glucoside	773	287,449,611	Unknown
Petunidin-3-*O*-caffeoylglucoside-5-*O*-glucoside	803	317,479,641	Unknown
Peonidin-3-*O*-caffeoylglucoside-5-*O*-glucoside	787	301,463,625	Unknown
Malvidin-3-*O*-caffeoylglucoside-5-*O*-glucoside	817	331,493,655	Unknown
Pelargonidin-3-*O*-caffeoylglucoside-5-*O*-glucoside	757	271,433,595	Unknown
Delphinidin-3-*O*-feruloylglucoside-5-*O*-glucoside	803	303,465	Unknown

Because varieties of *V. vinifera* only synthesize monoglucoside anthocyanins, whereas some other species in the genus Vitis, such as *V. coignetiae*, *V. rotundifolia*, *V. amurensis* and their hybrids usually have diglucoside anthocyanins in significant quantities as well, it is easily to detect the use of most hybrid grapes in the red wines by the presence of diglucosidic anthocyanins, especially the red French-American hybrids, in Appellation Control (AC) red wines [12,92]. This has played an important role in ensuring the usage of traditional grape varieties in certain French appellations, as well as in monitoring quality. However, this method still has its limitations, since some hybrids that are bred after many backcrosses to *V. vinifera* only produce 3-*O*-monoglucosides of anthocyanins, such some of the Seibel hybrids, for example S5455 [12]. Nowadays, triglucoside anthocyanins have not been found in young red wines [12].

### 2.2. Structure and Equilibria between Different Chemical Forms of Anthocyanins in Wines

**Figure 3:**
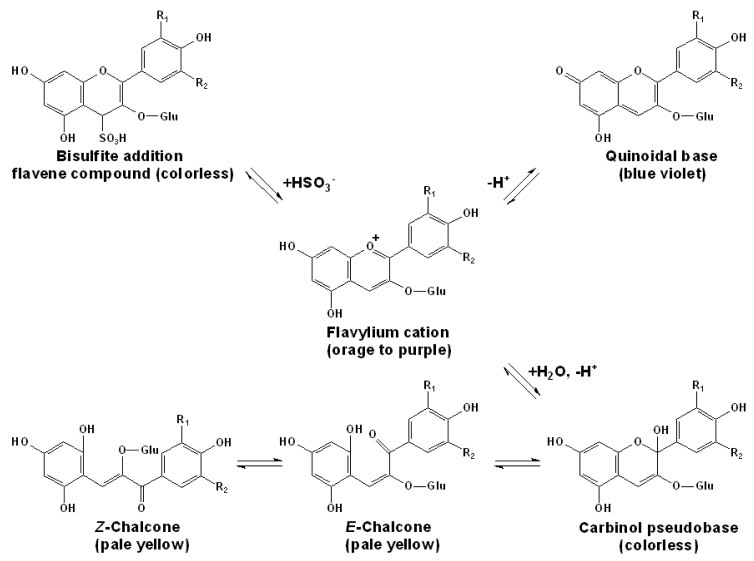
The pH-dependant equilibria among the various structural forms of anthocyanins in red wines [12,75,76,93,94,95,96,97]. The groups of R_1_ and R_2_ are listed in Figure 1.

In young red wines, where the monomeric anthocyanins are still present, they occur predominantly in a dynamic equilibrium among five major molecular forms, including the bisulfite addition flavene compound, the quinoidal base, the flavylium cation, the hemiketal or carbinol pseudobase and the chalcone (*cis-* and *trans-* forms), as shown in Figure 3 [12,75,76,93,94,95,96,97,98]. Each of these structure types can be distinguished by high-resolution proton NMR spectroscopy [75].

The flavylium cation state locates in the central part of the equilibrium and involves in two types of reactions, the acid-base reaction and the hydration. The conversion from the flavylium cation state to the quinoidal base state occurs by a very fast proton transfer, and the equilibrium between the flavylium cation state and the carbinol pseudobase state involves hydration and the following proton transfer in as relatively fast speed. The opening of the heterocycle and rearrangement of the carbinol pseudobase to form a chalcone occurs slowly, and the balance between *cis-* and *trans-*chalcone is quite slow and difficult to be changed. Because the speeds of these equilibriums are very different, they can be considered separately. The constants of hydration (*K_h_*) and proton-transfer (*K_a_*) are thermodynamic constants, which permit to calculate and define the distribution among anthocyanin forms under acidic young red wines conditions [75,93,94,95,96].

Among these states, the bisulfite addition flavene compound is the only one which is bonded to sulfur dioxide and the other four are free-forms. However, only the free anthocyanins in the flavylium state can contribute to the red color and the ones in the quinoidal base state can contribute to the blue color, both of which are only a small proportion in the total amount. At red wine pH (3.3–3.5), the equilibrium is largely towards the hemiketal state, which is colorless. Besides, the free anthocyanins in the reversed chalcones forms can offer some pale yellow color. Thus, the maximum visible absorption of young red wines at λ~520 nm principally arises from the flavylium ion and the quinoidal base forms [12,75,76,97,98].

The factors affecting the distribution among these forms and the color in young red wines are the pH value, the temperature and the amount of free sulfur dioxide. Low pH can increase the proportion of the flavylium state and retard the hydrolysis of the anthocyanins. As the pH rises, the concentration of the anthocyanins in the flavylium state and the color density decline rapidly. For example, at pH of 3.4–3.6, 20–25% of anthocyanins are in the colored flavylium forms, whereas at pH of 4.0, only 10% of anthocyanins are in such ionized state [12]. The maximum color loss is observed at pH of 3.2–3.5. Some studies also reported the methods to calculate the percentage of various forms of free anthocyanins according to the pH value, especially in wine with a pH between 3 and 4. Though the high pH can slightly increase the proportion of anthocyanins in quinoidal form which can contribute the blue-mauve color, it still significantly impairs the color density. Their color vary from mauve to blue at pH above 4, then fade to yellow in natural or alkaline medium [12,75,76,97,98].

However, the amount of the free sulfur dioxide is the most crucial factor that affects the color of young red wines. Sulfur dioxide can strongly bleach the free anthocyanins by the nucleophilic addition at the C4 position in the C ring of the anthocyanin in the flavylium cation in red wines, though such transformation is also reversible. At a pH of 3.2, about 96% of sulfuric acid consists of bisulfite anions that can react with the anthocyanins in the flavylium cation state to produce the colorless the bisulfite addition flavene compound. Thus, at a given concentration of sulfur dioxide, the color depends on the anthocyanins’ content: the higher the concentration, the more intense color [12,75,76]. 

### 2.3. Degradation of Free Anthocyanins

As mentioned, free anthocyanins in red wines are not particularly stable, and their concentration in red wines usually drops quickly during wine aging in barrels or bottles. After several years, although the wines remain red, there are almost no monomeric anthocyanins present. This is due to the polymerization or other modification reactions of the monomeric anthocyanins with other compounds in red wines, as well as the breakdown reactions of some of them [12,75]. 

Generally, the stability of the monomeric anthocyanins in red wines depends on various factors, such as their structure, concentration, solution composition, pH value of the wine, storage temperature and time, oxidation status, light exposure, the presence of other substances such as ascorbic acid, sugars, sulfites, cofactors and metallic ions [70,71,75,88,89,90,91,99,100,101,102,103,104,105]. Normally, anthocyanins seem to be more stable in acidic media at lower pH values than in alkaline solutions with higher pH values [99,101,103]. The stability of anthocyanins is greater at lower temperatures and also at higher concentrations [101,103,105]. Exposure to light promotes the degradation of anthocyanins in solutions [75,103]. The presence of ascorbic acid, sugar and their degradation products decreases the stability of anthocyanins [70,87,98,102,104].

When anthocyanin solutions are heated to a high temperature (such as during thermovinification), the solutions may lose their color quickly and irreversibly [12,103,105]. Moreover, the degradation rate increases with increasing temperature and juice concentration [108]. Ribéreau-Gayon *et al.* suggested that it could be a result of a shift in the equilibrium towards colorless chalcone form and subsequent breakdown of the carbon chain of the chalcone molecule [75]. However, glycoside hydrolysis may be another reasonable explanation [75,102].

On the other hand, in the acidic alcohol solutions, as in red wines, anthocyanins in the hydrated forms (chalcone or carbinol pseudobase) can react with *o*-diquinones generated by enzymatic or non-enzymatic oxidation readily to produce colorless and unstable chemicals, such as the corresponding phenolic acid and aldehyde [70,71,100,106,107,108]. In such conditions, oxygen and light seem to be the catalysts, and higher pH can also facilitate this reaction [101,102,103]. Because the adjacent hydroxyl groups of *o*-diaphanous are sensitive to oxidation, the malvidin-3-*O*-glucoside and peonidin-3-*O*-glucoside that do not possess *ortho*-positioned hydroxyl groups are comparatively more resistant to oxidation than cyanidin-3-*O*-glucoside during barrel aging of red wines [12,75,88,89,90,91,106,107,108].

The presence of ketones may be another factor that can cause degradation of anthocyanins. For example, in acidic solutions containing acetone, anthocyanins can react with it to form orange colored compounds. This phenomenon has been explained by various mechanisms, such as the hydrolysis the anthocyanins and the formation of dihydroflavonols, breakdown of the anthocyanins and the formation of benzoic acids, or condensation with acetone via the polarized double bonds [75].

## 3. Color Enhancement of Weak Complexes of Anthocyanins in Wines

In red grapes and young red wines, anthocyanins exist primarily as weak complexes, either with themselves termed self-association, or with other compounds, termed co-factors, resulting in the formation of copigments [109,110,111]. These are considered to be formed by processes that involve stacked molecular aggregation, which is primarily held together by hydrophobic interaction [12,110,112]. They can significantly increase color density (hyperchromic effect), and may affect color tint, a bathochromic effect giving a more purple hue to young red wines by provoking displacement of anthocyanin equilibria towards their colored forms, which can explain many questions of color expression in young red wines [113,114]. Furthermore, the better understanding of the self-association and copigmentation can also help us to predict the color attributes in young red wines from the phenolic profiles of red grapes [110,111].

### 3.1. Self-association of Anthocyanins

Self-association of anthocyanins is manifested by a positive deviation from Beer’s law, which can occur on ‘increasing’ the concentration of anthocyanins for one hundred times [109,110,111,115,116]. By both of the hydrophilic interactions between the glucose components of the corresponding anthocyanin molecules and the hydrophobic repulsion that take place between their aromatic nuclei and water, the vertical stacking of anthocyanin molecules in self-association complexes are promoted [12,109,110,111,115,116,117]. Thus, self-association can be recognized as a special form of copigmentation in which the copigments are anthocyanins themselves. Compared to the copigmentation among anthocyanins and other colorless co-factors, self-association might produce a hypsochromic shift, where the maximum absorption wavelength shifts toward the lower values [109,110,111,116].

Self-association can also influence the apparent hydration constant of the anthocyanins and subsequently modify the color of red wines [12]. In the previous studies, it was reported that the greater the degree of methoxylation in the B ring of the anthocyanin molecule, the greater was the extent of self-association [109]. In more recent research, it was reported that the self-association effect of malvidin-3-*O*-glucoside was thermodynamically favored over intermolecular interaction with any of the cofactors tested, suggesting that self-association of malvidin-3-*O*-glucoside cannot be neglected in young red wine. However, malvidin-3-*O*-(6′′-*O*-*p*-coumaryl)-glucoside did not show any color enhancement, suggesting that the *p*-coumaryl group prevents self-association [117]. Furthermore, self-association played a more important role in quantitative parameters (chroma) than in qualitative parameters (hue), indicating that self-association can intensify the color of the solutions and make them appear darker [109]. However, it has been suggested that the minimum concentration of anthocyanins for self-association to take place in the solutions needs to be greater than 1 mmol/L [109]. Furthermore, ethanol in the red wines will further limit the process of self-association, since such organic solvents can weaken the intermolecular hydrophobic interaction and counteract the complex’s formation [109]. Thus, the participation of the anthocyanin self-association in color expression of red wines with lower anthocyanin concentration could be limited. This appears to be particularly important in flower or berry coloration, rather than in young red wines [116].

### 3.2. Copigmentation of Anthocyanins

Copigmentation is one of the most significant factors accounting for a variable proportion of the color in red wines [2,75,110,111]. It refers to a solution phenomenon in which anthocyanin structures are formed within a given anthocyanin molecules, which is termed intramolecular copigmentation or between an anthocyanin and other colorless chemicals, termed intermolecular copigmentation [110,111,118,119,120,121,122,123]. The former structures are held by hydrophobic interaction (π-π stacking) of the polarizable planar nuclei of the colored forms of anthocyanins (both of flavylium cation and quinoidal base) with the aromatic residue of the same pigment [118,119,120,121]. In the latter type the π-π stacking occurs between the anthocyanin B ring and a planar B ring of a suitable molecule such as some phenolics [121,122,123]. More stable copigmentation complexes are seen where covalent bonds can be formed between aromatic acyl groups (especially the cinnamoyl residues) linked to the sugar moieties of anthocyanins and cofactors to provide additional stabilization effect [12,120,124]. Furthermore, it is also believed that copigmentation is not an immediate phenomenon, but it is established in a progressively way [125,126].

**Figure 4:**
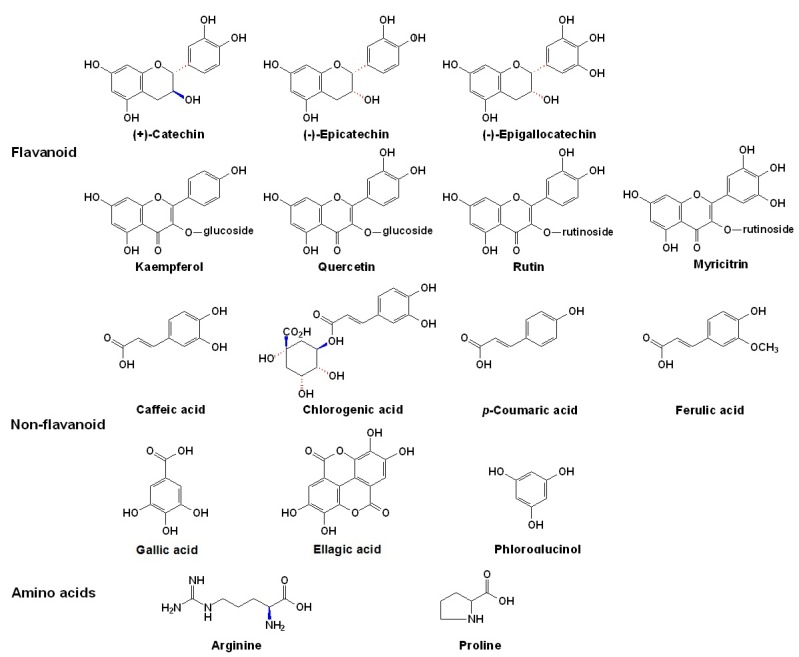
The structures of the major cofactors naturally occurring in young red wines [12,126,127,128,129,130,131,132].

Compared with self-association, copigmentation is more important in color modification in young red wines, promoting an increase in the maximum absorption wavelength of 5–20 nm typically (bathochromic effect), and causing a shift towards higher intensities (hyperchromic effect) [110,113,118,129,130]. The stacking of anthocyanin molecules in the copigmentation complexes produces a sandwich configuration, physically limiting water access to the chromaphore of the anthocyanins thereby limiting the formation of colorless hydrated forms (chalcone or carbinol pseudobase) [12,120,126]. Thus, copigmentation can result in greater color intensity of anthocyanin solutions than theoretically could be expected from the anthocyanin concentration and media pH effects [133]. Both colored anthocyanin states can also facilitate the interaction between the anthocyanin molecules and the cofactors. However, at the low pH value of young red wines, copigmentation primarily involves the monomeric anthocyanins in flavylium state [101,130,134,135]. Flavylium ions in the copigments do not participate in the overall anthocyanin equilibrium process so therefore copigmentation overall results in a greater proportion of the anthocyanins being in a colored stated; some in the copigmented state and the rest colored flavylium ions as could be predicted by the accepted equilibrium theory [12,120,126]. Furthermore, the hydrated anthocyanin forms that are not involved in copigmentation are more easily hydrolyzed into anthocyanidins and their glucose components [110,113]. The free anthocyanidins are both more sensitive to irreversible oxidative color loss and browning [70,75]. Thus, copigmentation also plays significant role in the color protection of anthocyanins, and copigments can exert a strong stabilizing effect on the color of the anthocyanins [121]. It is estimated that copigmentation can contribute about 30–50% of the color in young red wines [12,50].

The principal cofactors in young red wines are flavonoids and non-flavonoid phenolics, such as the flavonols, flavan-3-ols, oligomeric proanthocyanidins, cinnamic acids and hydroxycinnamoyl derivatives [110,113,127,128,129,130,131,132,133,134]. Normally, flavan-3-ols, such as (+)-catechin or (-)-epicatechin are recognized as powerful cofactors, which can form colored complexes most easily and intensely [112,126,127]. Some studies have reported that oligomeric flavan-3-ols have a much stronger copigmentation effect than the monomers, and even the ethyl bridged flavan-3-ols could act as strong cofactors [136,137]. One more recent study also reported that vinyl catechin dimers possessed a better copigmentation effect than procyanidin B3 [138]. But some other studies have reported that oligomeric flavan-3-ols, for example, procyanidin B2 are worse than the monomers, whereas flavonols are the best [125]. However, the concentrations and ratios of the cofactors used should be considered as these are important factors that can affect the results. It was also recommended that some hydroxycinnamic or cinnamic acids, such as caffeic acid, sinapic acid or ferulic acid, were more efficient in color enhancement than flavonoid phenolics at the same concentration level [129,139,140,141,142,143]. Considering that hydroxycinnamic acids are usually much lower levels than flavan-3-ols in red wines, flavan-3-ols are still the major cofactors. The variation of the relative proportion of these cofactors among different wines from different grape varieties, vintages and winemaking processes may cause distinct color differences between different wines [144,145,146,147,148].

Besides phenolic compounds in red wines, alkaloids, amino acids (mainly proline and arginine), some organic acids, polysaccharides, purines and metal cations can also participate in copigmentation [12,75,122,149,150,151,152,153,154,155,156]. In the latter situation, some anthocyanins with an *ortho*-dihydroxyl arrangement in the B ring, such as the glucosides of cyanidin, delphinidin and petunidin, and certain metal cations, such as Mg, Al, Fe (both of ferrous and ferric ions), Sn and Cu at levels of 10 mg/L can form colored complexes [75,110,111,154,155,156]. However, because malvidin-3-*O*-glucoside and its derivatives are the major anthocyanins in most *V. vinifera* red grapes and wines, anthocyanin-metal complexes are unlikely to play any significant role in the expression of red color [110,111]. The structures of the major cofactors in young red wines are illustrated in Figure 4, as shown above.

However, neither self-association nor copigmentation plays a crucial role in the coloration of rose wines, because of their low anthocyanin concentration, which are usually insufficient for the molecule aggregation. It is reported that the minimum concentration required for significant copigmentation is about 250 mg/L. However, the anthocyanin concentrations in rose wines are typically about 20–50 mg/L, which are dramatically lower than that required for copigmentation [12,157,158]. That is the reason why the blue or purple tones are absent in the rose wines.

Though copigmentation can noticeably affect the color of young red wines, there are various factors which influence the formation of these anthocyanin complexes, such as the pH values, cofactor and pigment structures, and their concentrations [101,130,134,135]. The suitable pH for copigmentation complex is around pH 3.5 [101,130,134,135]. Temperature is one of the most crucial factors. Cool fermentation and storage temperature can favor the process of copigmentation and retard the disassociation of the colored complexes. High temperatures, for example, heating grapes or must to improve color extraction (thermovinification) can destabilize the formation of self-association or copigmentation [101,135]. If insufficient phenolic or polyphenolic compounds are extracted from the pomace, this vinification technique may cause significant color loss, not only because of the poor copigmentation, but also the poor formation of polymeric pigments. Thus, even having the same anthocyanin concentration, the young red wines with low cofactors will show greater color loss than that would be predicted from their anthocyanin content, because of the enhanced dissociation of the colored complexes [12]. Thus, suitable vinification techniques are necessary for the red winemaking, such as maceration, or wood aging [12,147,159,160,161,162]. Alcohol also works against copigmentation by destabilizing the hydrogen bonding between anthocyanin aggregates, because it can disrupt the lattice-like interaction of water molecules and destroy the molecular stacking of the anthocyanins [134,163,164]. However, there are some literatures stating that ethanol seemed to enhance the hyperchromic shift. This was due to ethanol’s ability to facilitate the extraction of anthocyanins and cofactors from grapes [165]. Besides, UV irradiation has a strong degradation effect on the copigmentation complex, even greater than the treatment of heating at 80 °C [101,135].

## 4. Influence of Enology Practices on Anthocyanins in Wines

Although the categories and concentration of the monomeric anthocyanins in young red wines mainly depend on the grape variety, viticultural practices, extent of ripening and climatic conditions, wine making procedures, such as maceration conditions, the use of enzymes, fermentation temperature and conditions can also make a significant impact on the anthocyanin profile of red wines [69,73,148]. 

### 4.1. Effect of Maceration

Maceration is a term applied to several stages after crushing where the grapes skins are in contact with the grape must. Typically there can be three different maceration stages, pre-fermentation, fermentation and post fermentation [50].

Low temperatures maceration (5–15 °C) prior to fermentation, also known as ‘cold-maceration’ or ‘cold soak’, is one of the common alternative processes, which is designed to improve the extraction of pigments, tannins and aromas from the grape skins to the wine [166,167]. There are variable reports as to the impact of pre-fermentation maceration although many makers of Pinot Noir or other cultivars in particular claim improved color extraction and stability form using this “cold soak” step [163,168,169,170,171,172,173]. The extraction of these compounds takes place in the absence of ethanol because the low maceration temperatures prevent yeasts from starting the alcoholic fermentation. Consequently, it is frequently claimed that the resulting wines contains increased phenolic and anthocyanin content [163,172]. Interestingly, some new enological techniques, such as crushed grapes freezing with dry ice, can be recognized as modified ‘cold-maceration’, which can also lead to the wines with high color intensity and high anthocyanin content [5].

Maceration that occurs with fermentation is one of the most significant phases of red winemaking that affects the anthocyanin profiles in young red wines. It allows the diffusion of anthocyanins and other phenolic compounds from the solid parts of the grapes into the must or wine [50]. Extraction of anthocyanins from grapes during such maceration depends mainly on the maceration times and temperatures, frequency and mode of cap punching, alcohol and sulfur dioxide levels [147,174,175,176,177,178]. The active fermentation maceration temperature and duration has a very large impact on the content of anthocyanins in red wines [147,148,171,173,174,175,176,178]. Higher temperatures, such as 28 to 30 °C result in greater extraction than temperatures below 20 °C, with the maximum level of anthocyanin being reached at about 5 to 6 days [173,176]. Copigmentation continues to rise following alcoholic fermentation as does the polymerization process which begins almost with the onset of fermentation [148,163]. While the presence of ethanol facilitates anthocyanin and proanthocyanidin extraction in particular, it can decrease the level of copigmentation [134,163,164,165].

Normally, during traditional vinification, the concentration of monomeric anthocyanins will decreases after reaching a maximum level after a few days of fermentation because some of the extracted anthocyanins are adsorbed by yeast cell walls, precipitated with tartaric salts, and reduced by filtration and fining [175,179]. So the anthocyanin profile of one wine may be quite different to that of another wine, even when made from the same variety. The level of anthocyanins, monomeric, polymeric and copigmented in the final wine may not be highly correlated with the anthocyanin levels in the grapes [50,75].

Post-fermentation maceration plays a particularly important role in the pigment polymerization process and consequent color stabilization [184]. Special techniques such as thermovinification, flash heating and vacuum cooling of the crushed grapes, and carbonic maceration display different monomeric, copigmented and polymeric anthocyanin profiles, some of the differences being attributable to differences in proanthocyanidin extraction during the different processes [168,181,182,183,184].

Thermovinification is primarily used with cultivars with relatively low anthocyanin content or with diseased grapes such as reds with significant laccase content from botrytis [12]. The procedure involves heating intact or crushed grapes to 50–80 °C or exposing whole grapes to steam or boiling water for very short time (about or less than 1 min). Such treatment can destroy cell walls and membranes and inactivate laccase, resulting in the quick release of anthocyanins during subsequent maceration (45 °C for 6–10 h) without the initiation of enzymatic oxidation [2,185]. In the ‘flash-détene’ technique, the harvest grapes were heated quickly (80 °C) and then applied in high vacuum cooling (30 °C), which can result in a mechanical disruption of the grape tissue and promote the release of anthocyanins [186]. Thermovinification can dramatically increase anthocyanin extraction and the color intensity, whereas it can also lead to the loss of aromas and presence of strange odors [187]. However, thermovinification does not facilitate tannin extraction, and such technology is not good for the long term evolution of wine color, especially in the formation polymeric anthocyanin pigments [12,50,181,182]. Thus, it is normally used in the producing of wines for early consumption.

Carbonic maceration, used in the production of Beaujolais wines, occurs when whole grapes are held in a carbon dioxide rich environment thereby promoting anaerobic fermentation within the grape berry [184]. Compared to traditional maceration, carbonic maceration leads to very young light fruity red wines with lower anthocyanin content, mainly monoglucosides, and lower total phenols, but higher amounts of flavan-3-ols and oligomeric and polymeric proanthocyanidins [178,184,188].

The addition of pectolytic enzymes during maceration may also facilitate the extraction of anthocyanins into red wines, with a consequent increase the color intensity, although there are varying reports about the effectiveness of specific color extracting enzymes [5,147,148,160,177,178,179,180,181,182,183,184,185,186,187,188,189,190]. Pectolytic enzymes now are widely used in enology to improve juice yield and clarification by breaking down the pectins of the cell walls of the berries. In the case of red grapes this breakdown may assist with the release of anthocyanins from the skin cell walls, releasing anthocyanins and other phenolic compounds [178,190]. However, the purity of the enzyme preparation merits attention because some preparations have been found to contain β-glucosidases which can hydrolyze anthocyanins to their unstable aglycones, anthocyanidins, resulting in color loss [178]. Thus, some published works reported the use of pectolytic enzymes giving wines with higher anthocyanin content and a better visual density and hue, but others have reported no improvement or even poorer results [191].

The addition of enological tannins is another suitable way to improve wine color and its stability [148,177,192,193]. Though it will not help the extraction of anthocyanins from grapes, it will contribute usefully to the formation of polymeric pigmentations [192,193,194]. However, great care should be taken when using such commercial enological tannins to improve wine color and its stability, because the results depend a lot on the wine characteristics. Sometimes, they will cause the opposite effects and the resulted wine may lose their equilibrium and color stability, especially when the hydrolysable tannin is used [177,193]. Some of the variable response may lie in the fact that there are many different types of tannins and they vary in their preparation and composition. The addition of mannoproteins has also been tried but has not been found to maintain the extracted phenolic compounds in colloidal dispersion and did not contribute to color stability.

Some researchers have tried to use pulsed electric field (PEF) treatment to enhance the extraction of anthocyanins before maceration and fermentation, just after crushing grapes, which means it is really a pre-maceration technique [195,196,197,198]. The application of a PEF treatment led to freshly fermented model wines with higher concentration of anthocyanins and greater color intensity, showing better visual characteristics [195,196,197]. In a recent study, it was proposed that with the application of this technique, the produced aged wines showed better color characteristics [198]. The anthocyanin profiles of such freshly fermented model wines was similar to those of control wines, indicating that the change in skin cell membrane permeability by pulsed electric field treatment did not produce a selective effect on any particular anthocyanin [199].

### 4.2. Enhancement of Copigmentation

Since copigmentation contributes significantly to the color expression of non-polymerized anthocyanins and their stabilization in young red wines, it is reasonable for winemakers to investigate the techniques for enhancing the copigmentation process [110,111]. 

Adding cofactors into the must before alcoholic fermentation is one such approach, which should not only enhance the copigmentation process, but also facilitate the extraction of anthocyanins [140,144,200]. Some studies even used pre-harvest spraying of some cofactors, such as the flavonol rutin to improve the color of the red grapes and wines. However, so far field trials have not offered consistent results [201]. In the studies of Darias-Martín *et al.*, it was reported that the addition of (+)-catechin at 120 mg/L before fermentation resulted in 10% color enhancement after fermentation. They explained that such addition not only enhanced the copigmentation process, but also increased the formation of polymeric pigments from anthocyanin and the cofactors [200]. Hermosín-Gutiérrez *et al.* found that rutin could enhance both copigmentation and anthocyanin extraction, but the hydroxy-cinnamic acids (caffeic or *p*-coumaric acid) produced the opposite results. It is not easily to explain such totally opposite outcomes, but the varied grape cultivars, the degree of grape ripeness and vinification processes should be considered as the potential reasons [146]. Moreover, even the addition of the same cofactors before fermentation may lead to significant differences in red wine color according to the characteristics of their grape cultivars [144].

Cofermentation in which different red grape varieties are co-macerated and fermented together is another approach to copigmentation enhancement. Because different grape varieties have different concentrations of anthocyanins and cofactors, a complementary effect may be possible by the co-fermentation of different grape varieties to achieve a higher color density. In some research changes in the relative proportions of anthocyanins and hyperchromic shifts were observed, as well as anthocyanin self-association in the young red wines and the copigmentation process in the relatively aged red wine. It is likely that the proportions of different grape varieties and their viticultural history will influence the outcome of copigmentation in cofermentation [202]. 

### 4.3. Adsorption of Anthocyanins by Yeast Cell Walls

The yeast cell wall is made of mannoproteins that are bound to polysaccharides, glucanose and chitins [203]. The different polarities and the hydrophilic or hydrophobic nature of the cell wall polymers define their capacity to retain or adsorb different wine compositions, such as volatile compounds or pigments [204,205]. 

During vinification, different yeast strains can greatly influence the profile of anthocyanins and their derivatives [206,207]. Besides, some of the anthocyanins are adsorbed by the yeast cell walls and so are lost with the removal of the lees. Furthermore, the cell walls of different yeast strains can adsorb anthocyanins differently, and the qualities of anthocyanins adsorbed during fermentation by different yeasts are quite variable [205,208,209,210]. Detailed research has shown that acylated anthocyanins were more strongly adsorbed than non-acylated anthocyanins, whereas pyranoanthocyanins, such as vitisins were weakly adsorbed. Additionally, anthocyanins with greater degrees of methoxylation were adsorbed more strongly than the more hydroxylated ones, which suggested that adsorption involves a hydrophobic interaction [208,209]. Consequently, such adsorption will give an increase in yellow and a fall in blue, indicating that choice of yeast strain is quite important in red winemaking [208].

## 5. Conclusions

Extracted from grape berries, monomeric anthocyanins and their interaction with other phenolics or themselves contribute the main part of color in the young red wines. The anthocyanin composition in red wines depends not only on the original anthocyanin profile in grape berries, but also on the enological techniques applied. During the maturation and aging, the categories and the contents of these monomeric anthocyanins in red wines decline constantly, whereas red wines can still maintain an essentially red color, which mainly caused by the formation of the pyranoanthocyanins and the polymeric anthocyanin pigments [12,50,75].

The areas for future research into monomeric anthocyanins, their chemistry and behavior from grape growing though to wine making is still expanding and there is much to be done. Some of the fields requiring extensive investigation include:

(1) Comparative studies of the monomeric anthocyanin profiles of young red wines made from numerous different *V. vinifera* varieties and the influence of viticultural inputs on them.
(2) Profiling the monomeric anthocyanins in red wines from various non-*V. vinifera* varieties, as well as their intraspecific and interspecific hybrids.
(3) Identification of new monomeric anthocyanins in trace amounts in red wines.
(4) Copigmentation mechanism and enhancement practices, especially the discovery of more efficient cofactors.
(5) New enology practices to improve anthocyanin stability, formation of stable pigments, as well as total wine color.
